# The larval environment strongly influences the bacterial communities of *Aedes triseriatus* and *Aedes japonicus* (Diptera: Culicidae)

**DOI:** 10.1038/s41598-021-87017-0

**Published:** 2021-04-12

**Authors:** Elijah O. Juma, Brian F. Allan, Chang-Hyun Kim, Christopher Stone, Christopher Dunlap, Ephantus J. Muturi

**Affiliations:** 1 Department of Entomology, University of Illinois at Urbana-Champaign, 505 S. Goodwin Ave, Urbana, IL 61801 USA; 2 Illinois Natural History Survey, University of Illinois at Urbana-Champaign, 1816 S. Oak St, Champaign, IL 61820 USA; 3 Crop Bioprotection Research Unit, U.S. Department of Agriculture, Agricultural Research Service, 1815 N. University St., Peoria, IL 61604 USA

**Keywords:** Ecology, Microbial ecology

## Abstract

Mosquito bacterial communities are essential in mosquito biology, and knowing the factors shaping these bacterial communities is critical to their application in mosquito-borne disease control. This study investigated how the larval environment influences the bacterial communities of larval stages of two container-dwelling mosquito species, *Aedes triseriatus,* and *Aedes japonicus.* Larval and water samples were collected from tree holes and used tires at two study sites, and their bacteria characterized through MiSeq sequencing of the 16S rRNA gene. Bacterial richness was highest in *Ae. japonicus*, intermediate in *Ae. triseriatus*, and lowest in water samples. *Dysgonomonas* was the dominant bacterial taxa in *Ae. triseriatus* larvae; the unclassified Comamonadaceae was dominant in water samples from waste tires, while *Mycobacterium* and *Carnobacterium*, dominated *Ae. japonicus*. The two mosquito species harbored distinct bacterial communities that were different from those of the water samples. The bacterial communities also clustered by habitat type (used tires vs. tree holes) and study site. These findings demonstrate that host species, and the larval sampling environment are important determinants of a significant component of bacterial community composition and diversity in mosquito larvae and that the mosquito body may select for microbes that are generally rare in the larval environment.

## Introduction

Metazoan organisms serve as habitats for a variety of microbial taxa including bacteria, fungi, protists, and viruses. These microorganisms may provide critical biological functions in their hosts, and mutualistic interactions between hosts and their microbiota have been described^1–4^. In mosquitoes, the bacterial communities hosted by different mosquito species have been extensively described^5–9^, and studies on microbial functions has revealed that mosquito-associated bacterial communities, are essential for host nutrition, reproduction, development, defense against parasites and pathogens, and modulation of host immune function^10–15^. Mosquito guts host a low diversity of bacteria dominated by a few taxa most of which fall within the four broad phyologeneitic groups, namely Proteobacteria, Actinobacteria, Bacteroidetes, and Firmicutes^3,6–9,16–19^. Most of these are primarily acquired horizontally from the host environment including the larval environment, or in the case of adults, nectar and other dietary sources^10,20–24^. Thus, most of the bacterial communities that colonize mosquitoes are thought to be transient in nature. However, there is also evidence of transtadial transmission from larvae to adult stages among bacterial communities and their co-evolved mosquito host species, as well as vertical transmission from adult females to their offsprings in the case of intracellular bacteria such as *Wolbachia*^10,24–26^.

Bacterial diversity can vary substantially within and between mosquito species, influenced by a host of factors, including the mosquito life stage, dietary sources, feeding behaviour, and the mosquito host environment^16,27,28^. However, the contribution of the environment to the acquisition of bacterial communities is not yet fully understood. The interactions between bacterial commensals and their host mosquitoes has implications for mosquito fitness for disease transmission and therefore their application as tools for mosquito-borne disease control^29^. Thus, it is imperative to develop a better understanding of the factors that influence the succession pattern of bacterial communities in the host mosquitoes. Among the factors that have been shown to influence the composition and structure of mosquito microbiota include host genetics, species, age, sex, diet, stage of development, host sampling location and infection with parasites and pathogens^25,27,30–32^. The larval environment has also been shown to be an important factor in shaping the mosquito bacterial communities, however, in respect to two medically important mosquito species evaluated in this study, *Aedes japonicus* and *Aedes triseriatus*, the patterns of acquisition of bacterial communities from the larval environment has not been fully evaluated^1,6,16,33,34^.

Mosquitoes develop in a variety of natural and human-made aquatic habitats of diverse shapes and sizes. These include pools, ponds, stormwater catch basins, river and lake edges, rice fields, tree holes, indoor and outdoor water-holding containers, and discarded tires, among other habitats^35,36^. The bacterial composition and abundance in these aquatic larval habitats are driven mainly by the underlying environmental characteristics, precisely the organic detritus composition and their physical and chemical characteristics, which in turn may dictate the bacterial colonization patterns in immature and adult mosquito stages. Many of these habitats are fueled by organic detritus which provides a substrate for bacterial growth that is consumed by mosquito larvae and other macroinvertebrate consumers^37–42^. Different detritus types are known to support bacterial communities of varying composition and abundance, and detritus-mediated differences in physical and chemical characteristics of the larval habitats may cause further alterations in bacterial composition and structure in these habitats^38,43–47^. Stemflow and throughfall provide additional sources of inorganic nutrients such as nitrogen, phosphorus, and sulfates, which are key modifiers of the larval water chemistry parameters such as pH, dissolved oxygen, and conductivity, and thus play a key role in stimulating bacterial growth^40,48–50^. Water chemistry parameters vary significantly among larval habitats, seasons, and between locations^49^. Thus, detritus composition and the water chemistry characteristics of the larval habitats may have significant effects on bacterial communities that are acquired by mosquito larvae. However, we have limited understanding of how characteristics of the larval environment influence the composition and structure of mosquito-associated bacterial communities.

In this study, two container-dwelling mosquito species, *Ae. triseriatus* and *Ae. japonicus* were used to investigate how the larval environment influences bacterial composition and diversity in mosquito larvae. *Aedes japonicus* is an invasive species in the United States^51^. It is not considered a major vector of human pathogens although it has been found infected with several arboviruses including Japanese encephalitis virus (JEV)^52^, West Nile virus (WNV)^53^, La Crosse encephalitis virus (LACV)^54^ and Cache Valley virus (CVV)^55^. *Aedes triseriatus,* the primary vector of LACV, is the dominant tree hole mosquito species and co-exists with *Ae. japonicus* in woodland areas in the eastern and midwestern US^56,57^. Larvae of the two mosquito species occur in a variety of natural and man-made container aquatic habitats, but we specifically focused on tree hole and waste tire habitats, both of which are known to serve as ideal larval habitats for diverse mosquito species^35,40,58^. Tree hole and waste tire habitats differ markedly in the quantity, nature, and type of organic detritus they contain as well as in their chemical characteristics and detritus decomposition rates^37,38,40,49,58,59^. These differences are likely to influence the composition of resident bacterial communities and in turn, the pattern of bacterial community colonization in mosquitoes. This study tested the hypotheses that: (1) tree hole and waste tire habitats differ in their environmental characteristics and support different bacterial communities, (2) the bacterial composition of *Ae. triseriatus* and *Ae. japonicus* larvae reflect the bacterial communities of their aquatic larval habitats, and (3) water chemistry characteristics of the larval environment are important in shaping the bacterial community composition of the larval environment. This study found that host species, and the larval sampling environment are important determinants of bacterial community structure in mosquito larvae and that the mosquito body likely select for bacterial communities that are generally rare in the larval environment. The larval environment consisting of used tires and tree holes were characterized by differences in water chemistry parameters that also seemed to have a strong effect on the clustering of the dominant bacterial communities by habitat type (used tires or tree holes). Our findings add additional evidence of the importance of larval habitat to the assembly of internal bacterial communities of mosquito species neglected by previous research.

## Results

### Bacterial OTU taxonomic classification

**Figure 1:**
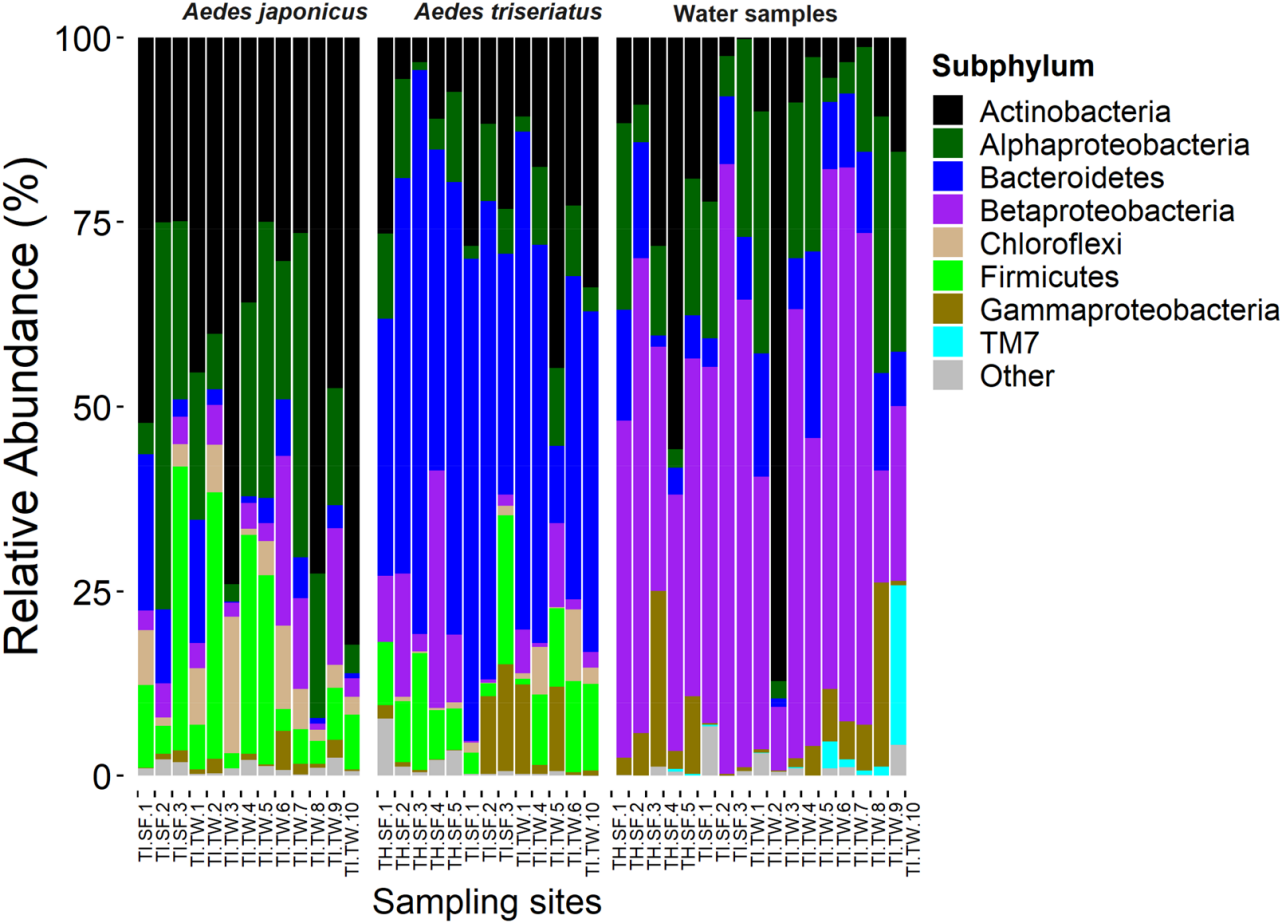
Composition of bacterial communities in mosquito and water samples collected from individual sampling sites at Trelease Woods and South Farms, Urbana, IL. Data represent taxonomic classification at subphylum level. Taxa with sequence abundance < 1% of total sequences were pooled together as “Other” in all the taxonomic ranks. *TI* used tire, *TH* tree-hole, *SF* South Farm, *TW* Trelease Woods.

MiSeq sequencing of the V3-V4 regions of the 16S rRNA gene from 276 samples (245 mosquitoes and 31 water) yielded 1,250,692 raw sequences (mean ± SE = 4531.49 ± 272.10 per sample). After quality filtering and rarefying the reads to an even depth of 1058 sequences per sample, 192 samples were retained. This data set consisted of 1,188,971 sequences (161 mosquitoes and 31 water) that were clustered into 918 bacterial OTUs. A total of 11 phyla, 25 classes, 91 families, and 123 genera, were detected. Eighteen of the 918 OTUs had an overall abundance of 1% or greater. The highest proportion of the sequences were from phylum Proteobacteria (34.2%). Other phyla included, Actinobacteria (29.1%), Bacteroidetes (23.4%), Firmicutes (9.0%), Chloroflexi (3.3%), with the rest of the phyla having taxonomic abundance of less than 1%. Water samples from most used tires and tree hole habitats were dominated by Betaproteobacteria, *Aedes triseriatus* samples were dominated by Bacteroidetes, while *Ae. japonicus* samples were dominated by Actinobacteria (Fig. 1).

At South Farms, Betaproteobacteria was dominant in water samples from used tires and tree hole habitats. *Aedes triseriatus* samples from used tire and tree hole habitats were dominated by Bacteroidetes. *Aedes japonicus* from used tire habitats were dominated by Actinobacteria and Alphaproteobacteria (Fig. 1).

At Trelease Woods, *Aedes triseriatus* samples from used tire habitats were dominated by Bacteroidetes and Actinobacteria. *Aedes japonicus* samples from used tires habitats were dominated by Actinobacteria and Proteobacteria (Alphaproteobacteria, Betaproteobacteria and Gammaproteobacteria). Water samples from used tire habitats were predominated by Proteobacteria. The rest of the taxa were represented in the three sample types at varying proportions but mostly < 10%. In general, only 1–2 major bacterial taxa were dominant in each sample type (Fig. 1).

**Figure 2:**
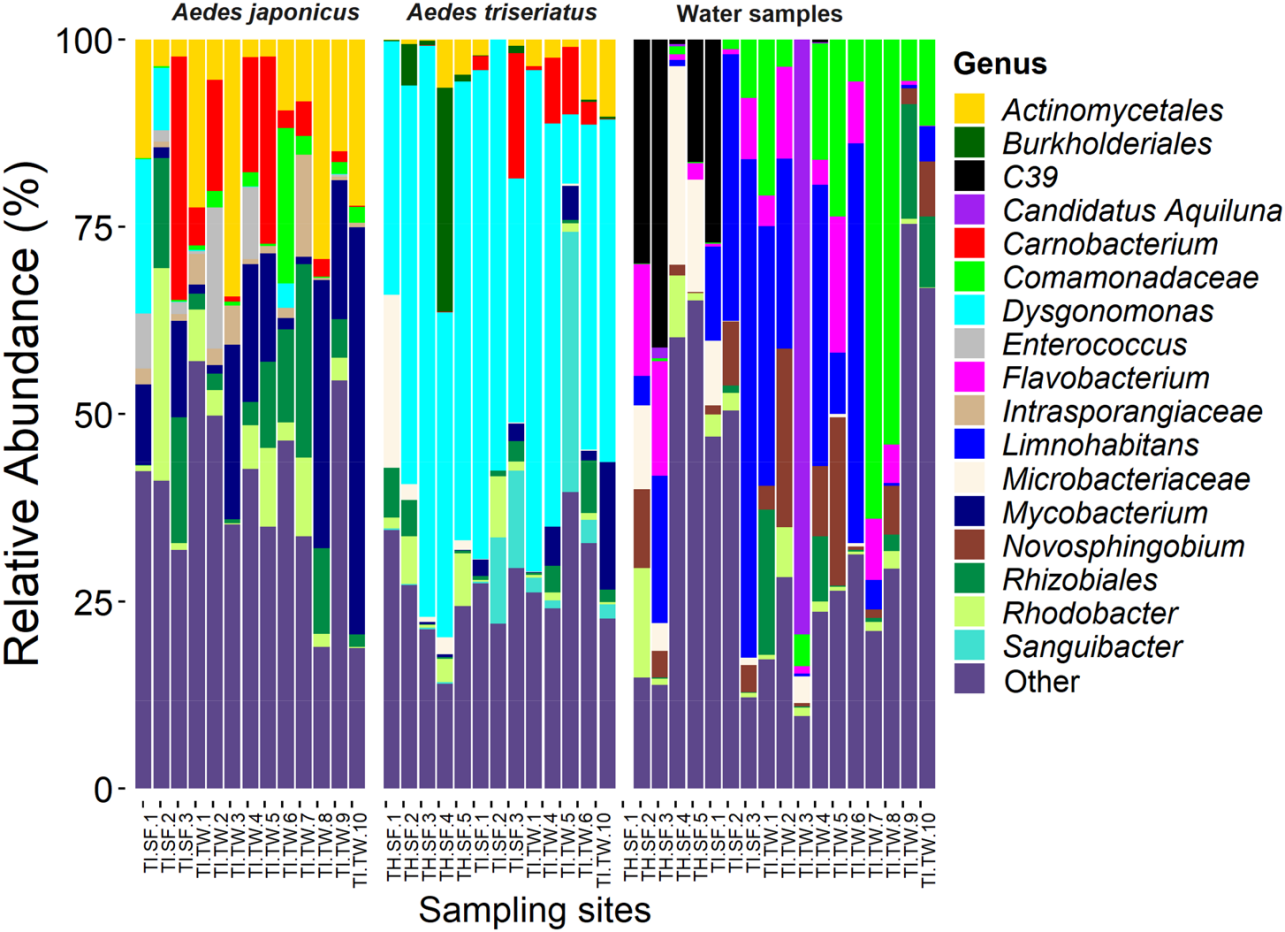
Composition of bacterial communities in mosquito and water samples collected from individual sampling sites at Trelease Woods and South Farms, Urbana, IL. Data represent taxonomic classification at the genus level. Taxa with sequence abundance < 2% of total sequences were pooled together as “Other” in all the taxonomic ranks. *TI* used tire, *TH* tree-hole, *SF* South Farm, *TW* Trelease Woods.

The most abundant bacterial OTUs overall were associated with the genus *Dysgonomonas* (20.0%), an unclassified Actinomycetales (7.5%), *Mycobacterium* (7.0%), an unclassified Rhizobiales (6.1%) and an unclassified Comamonadaceae (4.4%). *Dysgonomonas* were dominant in *Ae. triseriatus* samples from most of the used tire and tree hole habitats. The unclassified Actinomycetales, *Mycobacterium* and the unclassified Rhizobiales occurred in disproportionally high abundance in *Ae. japonicus* samples from used tire habitats, both at South Farms and Trelease Woods. The unclassified Comamonadaceae was the dominant bacterial OTU in water samples from most of the used tire habitats at Trelease Woods, while the taxon *Limnohabitans* was dominant in used tire habitats at South Farms (Fig. 2). Some tree hole habitats at South Farms were associated with relatively high proportions of the bacterial peptidase family C39. Bacteriocin-processing peptidase are cysteine peptidases common in both gram-positive and gram-negative bacteria and are responsible for the maturation of bacteriocins^60^ (Fig. 2).

### Bacterial diversity within mosquito and water sample treatments

**Figure 3:**
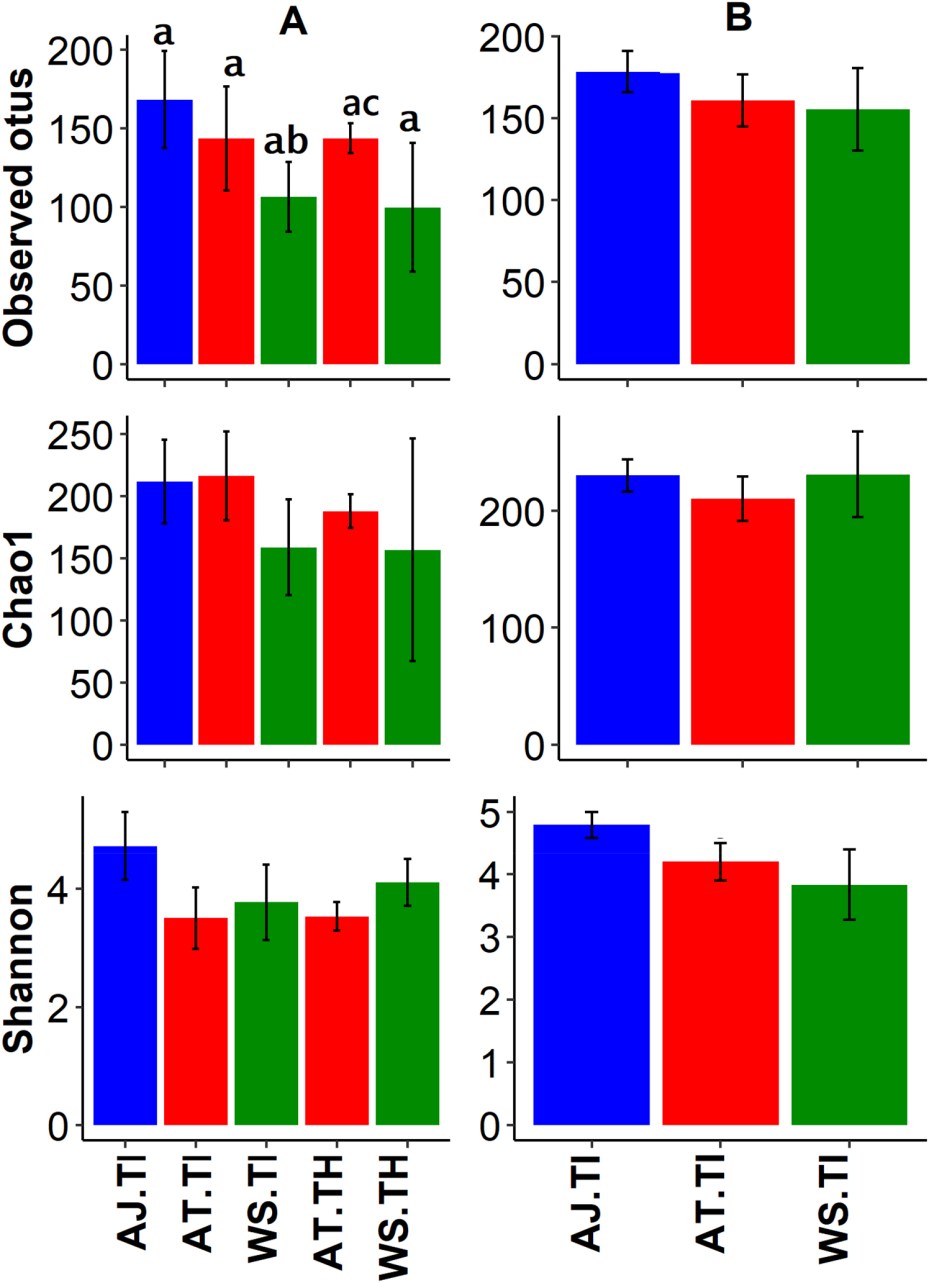
Bacterial OTU diversity (mean ± SE) observed combinations comprising of mosquito and water samples partitioned by habitat type and study site. (**A**) South Farms samples; (**B**) Trelease Woods samples. Combinations included: *AJ Aedes japonicas*, *AT Aedes triseriatus*, *WS* water samples, *TI* used tires, *TH* tree holes. Different letter symbols represent statistical significance.

Rarefaction curve analysis (an interpolation and extrapolation method for comparing species richness based on samples of equal sizes) recovered majority of the bacterial OTUs by the sequencing depth coverage of 1058 sequences (Fig. S1)^61^. Chao1 estimator (an abundance-based diversity index for estimating rare species) revealed that up to 75.0% ± 0.03 (mean ± SE) of the expected bacterial OTUs were detected^62^. Rarefaction analysis showed that *Ae. japonicus* from used tires from Trelease Woods had the highest OTU richness while water samples from tree holes at South Farms recorded the lowest OTU richness (Fig. S1). Alpha diversity analysis revealed variations in OTU richness and diversity among sample types. At South Farms, the observed number of OTUs were significantly higher in *Ae. japonicus* from used tires compared to water samples from used tires, and water samples from used tires and *Ae. triseriatus* from tree holes (Kruskal–Wallis chi-squared = 17.85, df = 4, *p* = 0.001) (Fig. 3A, Table S1). At Trelease Woods, there were no significant differences in the bacterial OTU numbers observed in *Ae. japonicus* from used tires compared to water samples from used tires (Observed OTUs: Kruskal–Wallis chi-squared = 5.15, df = 2, *p* = 0.08) (Fig. 3B, Table S1).

Overall, 537 (57.6%) OTUs were shared between mosquito larvae and water samples. More OTUs (36.0%) were observed in larval samples compared to water samples (6.4%) (Fig. S3A). At South Farms, more OTUs were unique to *Ae. triseriatus* from tree holes (62.5%) compared to the number shared between *Ae. triseriatus* and water samples (23.4%) (Fig. S3B). *Aedes japonicus* samples from used tire habitats had more bacterial OTUs unique to them (20.2%) compared to those shared between *Ae. triseriatus, Ae. japonicus* and water samples (19.1%) (Fig. S3C). At Trelease Woods, more OTUs were shared (32.0%) between *Ae. triseriatus, Ae. japonicus* and water samples from used tire habitats compared to OTUs that were unique to either sample type (Fig. S3D). When all samples from used tires from both South Farms and Trelease Woods pooled together, more OTUs were shared between the three sample types (41.7%) compared to the number of unique OTUs for each sample type (Fig. S3E). Generally, *Ae. japonicus* had more OTUs compared to *Ae. triseriatus* (Fig. S3C–E). Pooled *Ae. triseriatus* samples from both study sites revealed that *Ae. triseriatus* samples used tire habitats at Trelease Woods had more OTUs unique to them (34.6%) compared to shared OTUs (13.7%) (Fig. S3F). Pooled *Ae. japonicus* samples from used tire habitats from both study sites revealed more shared OTUS (49.0%) and samples from Trelease Woods had more OTUs compared to samples from South Farms (Fig. S3G). Pooled water samples from both study sites showed water samples from used tire habitats at Trelease Woods had more unique OTUs (35.0%) compared to shared OTUs (8.2%) (Fig. S3H).

### Bacterial composition between mosquito and water samples across individual sampling sites

**Figure 4:**
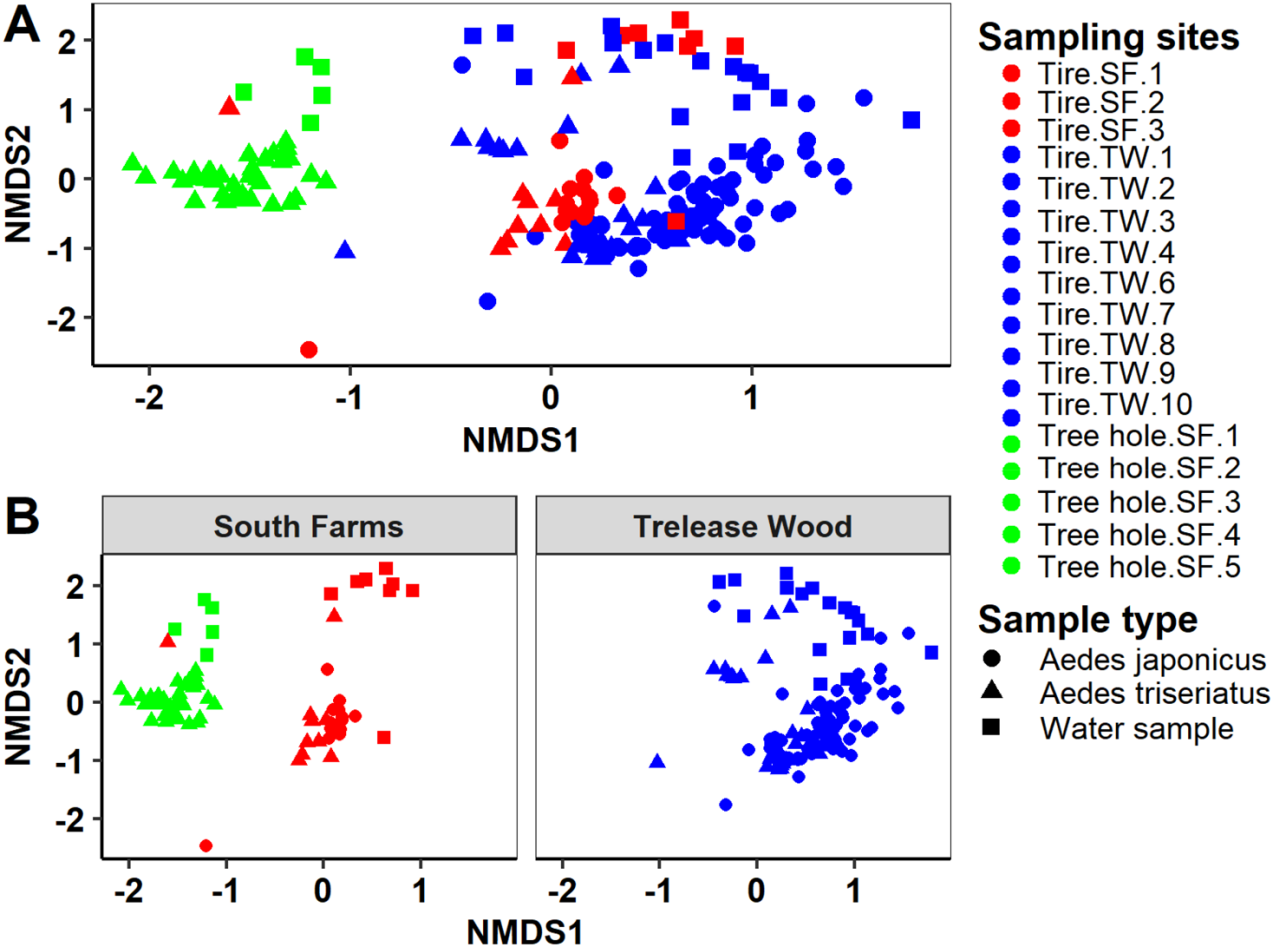
NMDS using Bray–Curtis distance matrix comparing bacterial communities between *Aedes japonicus, Aedes triseriatus* and water samples by individual sampling sites. (**A**) Combined plot; (**B**) bacterial OTU data partitioned by habitat type. *SF* South Farms, *TW* Trelease Woods.

PERMANOVA analysis revealed significant differences in the bacterial composition by mosquito species and water samples (Tables 1, 2), and between *Ae. japonicus*, *Ae. triseriatus*, water samples, at the individual sampling sites at South Farms and Trelease Woods, respectively (Tables 3, 4). Similarly, PERMDISP analysis revealed significant differences in bacterial composition by mosquito species (*betadisper*: F = 4.43, *P* = 0.018), and between *Ae. japonicus*, *Ae. triseriatus*, water samples, at the individual sampling sites level at South Farms (*betadisper*: F = 4.78, *P* = 0.001); but not at Trelease Woods (*betadisper*: F = 1.70, *P* = 0.102), indicating dispersion effect on the bacterial composition of mosquito and water samples at South Farms, but no dispersion effect on bacterial composition at Trelease Woods. Post-hoc analysis using multiple pairwise comparison of the individual sampling sites revealed significant differences in bacterial community composition between *Ae. triseriatus, Ae. japonicus* and water sampling from most of the individual sampling sites at South Farms and Trelease Woods, respectively (Tables S2–S4). NMDS plots using Bray–Curtis distance matrix revealed several distinct clusters separating the experimental samples by host species and individual sampling sites (Figs. 4, S2).

**Table 1:** PERMANOVA results based on Bray–Curtis dissimilarities evaluating differences in the bacterial community composition between sample type (*Aedes japonicus*, *Aedes triseriatus*, and water samples) by individual sampling sites.

Treatment	Df	SS	Mean SS	F. model	R^2^	*Pr*(> F)
Sampling habitats	17	39.33	2.31	16.27	0.48	0.001
Sample type	2	6.51	3.26	22.89	0.08	0.001
Sampling habitat: sample type	23	14.81	0.64	4.53	0.18	0.001
Residuals	149	21.19	0.14		0.26	
Total	191	81.83			1.00	

*P*-values are based on 999 permutations.

**Table 2:** PERMANOVA results based on Bray–Curtis dissimilarities evaluating differences in the bacterial community composition between *Aedes japonicus*, *Aedes triseriatus*, water samples.

Treatment	Df	SS	Mean SS	F. model	R^2^	*Pr*(> F)
Sample type	2	11.25	5.62	15.05	0.14	0.001
Residuals	189	70.59	0.38		0.86	
Total	191	81.83			1.00	

P-values are based on 999 permutations.

**Table 3:** PERMANOVA results based on Bray–Curtis dissimilarities evaluating differences in the bacterial community composition between *Aedes japonicus*, *Aedes triseriatus*, water samples at each individual sampling site at South Farms study location.

Treatment	Df	SS	Mean SS	F. model	R^2^	*Pr*(> F)
Sampling sites	7	14.12	2.02	9.82	0.50	0.001
Residuals	68	13.95	0.21		0.50	
Total	75	28.07			1.00	

P-values are based on 999 permutations.

**Table 4:** Permanova results based on Bray–Curtis dissimilarities evaluating differences in the bacterial community composition between *Aedes japonicus*, *Aedes triseriatus*, water samples at each individual sampling site at Trelease Woods study location.

Treatment	Df	SS	Mean SS	F. model	R^2^	Pr(> F)
Sampling sites	8	16.31	2.04	7.43	0.37	0.001
Residuals	102	27.95	0.27		0.63	
Total	110	44.26			1.00	

*P*-values are based on 999 permutations.

### Association between bacterial structure and water chemistry parameters

**Figure 5:**
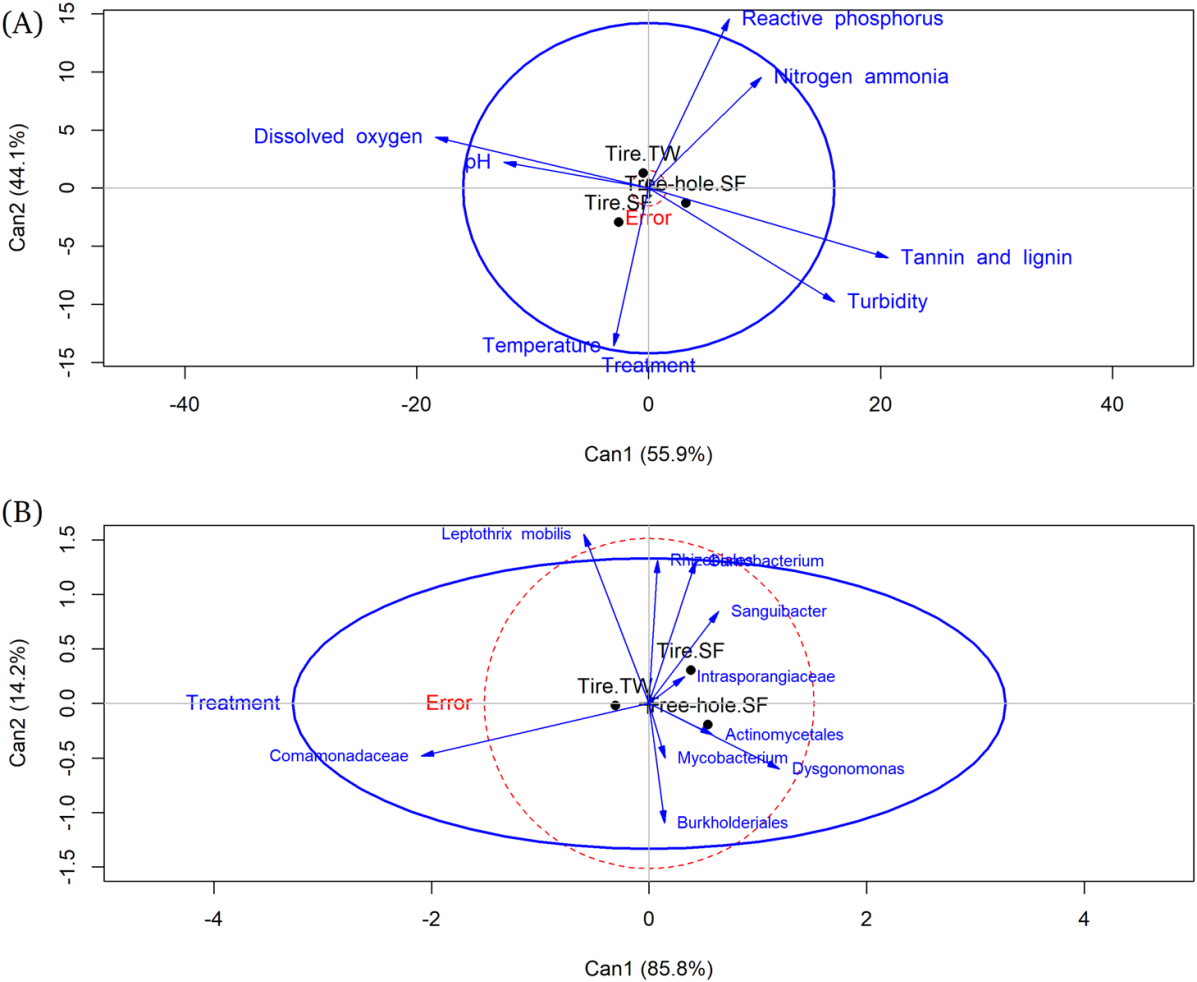
Canonical discriminant analysis of the correlation between water chemistry parameters and larval environment type (**A**); and the correlation between dominant bacterial OTUs in the water samples and larval environment type (**B**). The lines represent the h-plot of important water chemistry parameters, or dominant OTUs, the points represent the means for the larval environment type. The length of the line of the variable in the plot is proportional to its importance in separating the data into the three habitat types.

Water chemistry characteristics of the larval environment varied substantially depending on the habitat type and the study site. Tree hole habitats at South Farms were characterized by low pH, moderate temperature and reactive phosphorus, and high conductivity, TDS, salinity, tannins, and lignin, turbidity, and nitrogen-ammonia (Fig. S4). Used tires at South Farms were characterized by high pH, temperature and dissolved oxygen, and low TDS, conductivity, salinity, nitrogen-ammonia, reactive phosphorus, tannins and lignin and turbidity. Used tire habitats at Trelease Woods registered high pH and reactive phosphorus, moderate temperature, dissolved oxygen, and nitrogen-ammonia; and low conductivity, TDS, salinity, tannins and lignin, and turbidity (Fig. S4). Canonical discriminant analysis correctly classified 96.9% of used tire habitats at South Farms, 90.3% of used tire habitats at Trelease Woods and 90.7% of tree hole habitats at South Farms, based on water chemistry parameters. The length of the line of the variable in the plot is proportional to its importance in separating the data into the three habitat types (Fig. 5A)^63^. Reactive phosphorus and nitrogen ammonia were most important in separating used tires at Trelease Woods on the second discriminant function (Table S5, Fig. 5A) Tannin and lignin were important in separating tree holes at South Farms on the first discriminant function. The first two discriminant functions were statistically significant for each larval habitat type (Table S5). Regarding the dominant bacterial OTUs, canonical discriminant analysis correctly classified 56.3% of the three larval habitats (used tire habitats at South Farms, used tire habitats at Trelease Woods, tree hole habitats at South Farms) based on the dominant bacterial OTUs. *Mycobacterium* and Rhizobiales were the most dominant bacterial OTUs separating used tire habitats at South Farms on the first discriminant function. *Dysgonomonas* and an unclassified Burkholderiales were the dominant bacterial OTUs separating tree holes at South Farms on the first discriminant function (Table S6, Fig. 5B). The unclassified Comamonadaceae was the most dominant bacterial OTU separating used tire habitats at Trelease Woods on the first discriminant function. None of the discriminant functions were statistically significant for any of the three larval habitat types (Table S6).

## Discussion

The focus of this study was to understand the relative role of the larval environment in shaping the composition and diversity of mosquito-associated bacterial communities. To achieve this, we characterized and compared the bacterial communities of late fourth instar larvae of *Ae. triseriatus* and *Ae. japonicus* and water from their tree hole and waste tire habitats at two study sites in Urbana, Illinois. The bacterial community composition differed between the two mosquito species and was also influenced by the larval sampling habitats. These findings improve current knowledge of the factors that shape bacterial composition and diversity in mosquitoes and provide a framework for understanding how these factors may impact mosquito-microbe interactions that are relevant to disease transmission and control.

The observation that *Ae. japonicus* and *Ae. triseriatus* larvae harbor distinct bacterial communities is consistent with previous reports that host species is an important factor shaping the mosquito bacterial communities^8–10,18,64^. The factors responsible for host-specific variation in larval bacterial communities of the two mosquito species were not quantified in this study, but several factors are likely to explain these differences. First, larvae of the two mosquito species exhibit different foraging behavior, which could potentially expose them to unique bacterial communities. *Aedes triseriatus* larvae are both filter-feeders in the water column and browsers on microorganisms attached to the organic substrates such as leaf detritus or on the walls and bottom of container habitats^40,65,66^. In contrast, the foraging behavior of *Ae. japonicus* larvae vary depending on the container type, although they mostly spend their time browsing along the upper water edge of the container^67^. This likely expose them to distinct bacterial assemblages occupying different niches in the container environment. It is also possible that larvae of the two mosquito species are exposed to similar bacterial communities, but the gut system of each mosquito species is selective in the type of microbes that colonize and persist in them. This may occur due to species differences in the host immune system, inherent genetic differences and other physiological conditions such as gut pH, redox state, digestion and assimilation rates of the ingested bacterial communities, and presence of other microorganisms^68–72^. Pang et al.^72^ have shown that *Aedes aegypti* and *Culex pipiens pallens* differ in their pattern of bacterial survival and colonization due to the presence of C-type lectins, a large group of proteins that modulate metazoan immune mechanisms by binding directly to microbes^72^. Other studies have shown that the ability of bacteria to colonize mosquito guts may be influenced by whether they are transient or not^68,73^. Some bacterial communities in this study may have been transient, and thus preferentially eliminated, accounting for differences in the bacterial community composition between the two species^68^. Additionally, the ability of mosquito species to regulate oxidative stress determines the bacterial succession patterns that manifest in the mosquito guts^21^. These differences also could manifest differently between *Ae. japonicus* and *Ae. triseriatus* in this study, and possibly contributed to the observed differences in bacterial composition between the two species.

*Dysgonomonas* spp*.* was by far the dominant bacterial genus in *Ae. triseriatus* samples, while no clear dominance of any bacterial taxon was observed in *Ae. japonicus*. The strong association between some bacterial taxa and their mosquito hosts has been linked to ‘colonization resistance’ phenomenon in which the dominant taxa, or the bacterial taxa that colonize their mosquito hosts early on in their life history, create an environment that excludes, or dictates the pattern of successive bacterial colonization, either through competition for resources, or production of inhibitory molecules^3,74,75^. However, we do not have evidence to suggest that *Dysgonomonas* colonized larval samples earlier compared to other bacterial communities. Possibly on account of priority effects it may be that in this study *Dysgonomonas* were superior competitors perhaps through niche preemption or modification, and therefore were readily assimilated. *Dysgonomonas* therefore, dictated the pattern of subsequent colonization by successive bacterial communities^76^. *Dysgonomonas* was also detected in water samples in low abundance and was also in low abundance in *Ae. japonicus* samples from waste tires in Trelease Woods, and present in low abundance in *Ae. japonicus* samples from waste tires at South Farms. This finding may suggest that either *Dysgonomonas* is uniquely adapted to colonize and thrive in *Ae. triseriatus* larval tissues or that members of this genus occupy other ecological niches within the container habitats other than the larval water where *Ae. triseriatus* may encounter them, such as leaf surfaces or container walls.

Interestingly, *Dysgonomonas* was not detected in adult females of *Ae. triseriatus* and *Ae. japonicus* collected as pupae at the same study sites (Muturi et al*.* 2016a). We suspect that since *Dysgonomonas* was detected in water samples, albeit at low abundance, *Ae. triseriatus* and *Ae. japonicus* larvae potentially acquired this taxon from the larval environment, but the majority are possibly lost during molt from larvae to pupae rendering them undetectable in adult samples. Future studies characterizing the bacterial communities of *Ae. triseriatus* or *Ae. japonicus* across life stages could help test this hypothesis. Both facultative and obligate *Dysgonomonas* adapted to the anoxic mosquito gut environment have been described in both larvae and adults of several mosquito species, including *Ae. albopictus, Anopheles gambiae s.l., Culex tarsalis*, and *An. stephensi*^5,16,17,20,25,28,64,77^. In termites, *Dysgonomonas* have been reported to mediate vitamin B_12_ synthesis^78^. However, the role of *Dysgonomonas* in mosquito biology remains to be described and warrants additional attention in future studies.

Few studies exist that have characterized the bacterial communities of *Ae. japonicus* and *Ae. triseriatus*^9,18,79^. Previous research reveals variation among studies in the dominant bacterial taxa of *Ae. triseriatus* and *Ae. japonicus*, possibly due to environmental and seasonal differences. A study with larval samples collected from different sites in Champaign, Illinois, revealed the dominance of the bacterium *Bacillus cereus* in both *Ae. triseriatus* and *Ae. japonicus*^79^*.* A related study with adult samples reported the dominance of the bacterial genera *Gluconobacter* and *Wolbachia,* to a lesser degree in *Ae. japonicus*, and the bacteria genera, *Morganella* and *Gluconobacter* in *Ae. triseriatus*^9^*.* Another study with adults of *Ae. japonicus* and *Ae. triseriatus* revealed the dominance of *Paenibacillus* and *Polynucleobacter* in newly emerged *Ae. japonicus* and *Ae. triseriatus* samples, respectively^18^. These studies point to the possibility that variations in bacterial community composition among similar species but from different studies may also also be influenced by differences in bacterial colonization pattern in the larvae and adult stages^80^.

An unclassified Comamonadaceae dominated water samples from used tire habitats at Trelease Woods, while the genus *Limnohabitans* (Betaproteobacteria, Comamonadaceae) was dominant in used tire habitats at South Farms. The family Comamonadaceae is a group of gram-negative aerobic bacteria that metabolize organic acids, including amino acids^81^. They have previously been isolated from mosquito larvae and their larval environment as well as in the soil and water from natural and industrial environments^74,81–85^. They also have been reported as part of the core bacterial communities of adult mosquito species such as *An. gambiae* and *An. coluzzii*, pointing to potential transtadial transmission among conspecifics, and/or horizontal acquisition from the larval environment^10,16,19,21,25,31,33,86,87^. Other studies report the dominance of the members of Comammonadaceae in aquatic habitats without mosquito larvae relative to larval habitats colonized by mosquito larvae, suggesting that members of this family likely serve as a major component of larval food resources^83^. The genus *Limnohabitans* is a member of aerobic anoxygenic phototrophic bacterial communities with a cosmopolitan distribution in fresh water—and other aquatic environments^88,89^. This genus occurred in our mosquito samples in insignificant fractions, indicating that although it may be acquired horizontally in the larval environment by mosquitoes, it is readily digested and excreted, or it is poorly adapted to colonizing the anoxic mosquito gut environments. Studies characterizing *Limnohabitans* in other mosquito bacterial studies are also unavailable.

*Aedes triseriatus* larval samples from tree hole and used tire habitats had distinct bacterial communities suggesting a strong influence of larval sampling site on mosquito bacterial communities. Discriminant analysis identified different water chemistry parameters that characterized used tire and tree hole habitats, that also seemed to have a strong effect on the clustering of the dominant bacterial communities by habitat type (used tires or tree holes). The role of the larval environment in exerting an influence on the mosquito bacterial composition has been reported previously^16,24,28,30,33,90^. For example, Kim et al*.*, found strong variations in the bacterial communities within conspecifics (*Ae. japonicus* and *Ae. triseriatus* adults) sampled from different sites, but less variation between different genera (*Culex* and *Aedes*) sampled from the same site. Discriminant function analysis results revealed that water from tree holes was highly turbid and had higher concentrations of tannins and lignins. Conversely, used tire habitats were characterized by higher concentrations of reactive phosphorus and nitrogen ammonia. The differences in the physical and chemical characteristics of the two habitat types were likely due to differences in detritus types. Previous studies have shown that tree holes and used tire habitats vary significantly in their detritus composition, and these differences may have a dramatic effect on their bacterial composition^91^. Tree holes at South Farms are naturally more permanent compared to the used tires sampled in this study. Although the detritus composition of the used tire and tree hole habitats were not quantified, some studies suggest that tree holes generally harbor high abundance of both plant and animal detritus accumulated over multiple years compared to used tires, and thus are likely to structure the bacterial community differently to the transient used tire habitats^91,92^. The detritus is mostly composed of senescent plant parts, primarily terrestrial leaf detritus, and some input of animal detritus that provide substrate for bacterial growth and hence shape the bacterial community composition of the container habitats^48,91,93,94^. Additionally, during the sampling period, it rained at least once every week, and this is likely to have increased stemflow into the tree-holes. Stemflow provides soluble nutrient input that stimulates the growth of diverse bacterial communities in the tree-hole habitats^40,49,95^. In contrast, the used tire habitats were more ephemeral having been established and sampled two weeks into the duration of the sampling period, and therefore, were likely to harbor a different mix of detritus leading to different sets of bacterial communities between the used tire and tree hole habitats. We also observed differences in the physical and chemical profile between the used tire and tree hole habitats which may have additionally driven the difference in the bacterial assemblages of the two habitat types.

Some bacterial OTUs detected in mosquito larvae were not found in the water samples, and overall, fewer bacterial OTUs were observed in the water samples compared to the mosquito samples. This is contrary to previous studies showing that the larval environment harbors higher bacterial OTU richness compared to the mosquito larvae^1,21,29,74,79^. These findings suggest that some bacterial communities in mosquito bodies are likely transmitted vertically or horizontally via conspecifics or that some of the microbes occur in very low abundance in the larval environment that could not be detected by our methods. Additionally, bacterial communities in the larval environment occupy different niches including the surface microlayer, the sub-surface, and substrates such as the container walls and detritus surfaces^35,37,82,96^. It is possible that *Ae. triseriatus* and *Ae. japonicus* larvae spent most of their time browsing on either the leaf surfaces or container strips that possibly contained higher bacterial richness compared to the water column. This is supported by previous reports showing that the larvae of container-dwelling mosquitoes, such as *Ae. triseriatus*, prefer to graze on the leaf substrate and container surfaces over the water column^37,65,96,97^. We also agitated the larval water prior to sampling disrupting the surface microlayer and the sub-surface, possibly reducing the recovery rates of bacterial communities occupying these niches. There is also a possibility that the 40 mL of water from the larval habitat used in the bacterial analysis was not sufficient to capture a representative sample of the bacterial communities in the larval habitats. However, this volume falls within the standard volumes that have been used in previous studies, but which have shown higher bacterial richness in water samples relative to mosquito samples^16,79^.

Our study is limited by the use of whole-body mosquito larvae as opposed to midgut samples. Therefore, not all the mosquito bacterial communities that were characterized in this study could be assigned to the gut. Apart from the midgut, mosquito bacterial communities have been isolated from other organs of the mosquito body such as the ovaries and salivary glands, among others^98–101^. However, it is well established that most mosquito bacterial communities are concentrated in the midgut and are also the most studied group^4,102^. Bacterial communities also colonize the external surfaces of mosquito larvae, creating the possibility that some of the bacterial communities that were isolated in our study could be from the external surface. We thoroughly sterilized our whole-body mosquito larval samples according to previously used protocols^8^, reducing the possibility that the bacterial communities characterized in our whole-body larval samples could have included external body surface bacterial communities. Additionally, the bacterial communities isolated in our larval samples are similar to those previously isolated in other studies with midgut samples^4,103^. Additionally, sample coverage was assessed using rarefaction curves at a maximum read depth of 1058 sequences. The rarefaction curves did not plateau fully at this sequencing depth, indicating that higher bacterial richness and diversity could have been recovered with additional sequencing at higher depth. However, rarefying at higher sequence count in this study would have resulted in too many samples being discarded, hence the threshold utilized.

Understanding the factors that shape the bacterial composition and diversity in mosquitoes is an essential step in their manipulation for bacterial-based mosquito-borne disease control. Our study has shown that while the host environment is an important driver of mosquito bacterial composition, the bacterial composition does not fully reflect the environmental bacterial assemblages. Whereas this study suggests that mosquitoes may select for the suite of bacterial communities that colonize, we are unable to fully infer the source of the higher numbers of bacterial OTUs detected in the mosquito samples compared to the water from the larval habitat. This leaves a gap in our understanding of the source of the bacterial communities that colonize mosquito bodies and additional studies are needed to address this knowledge gap. Future studies may evaluate in controlled experimental conditions the unknown stress factors regulating bacterial diversity and composition in the larval environment, and thus subsequently influencing the bacterial succession pattern of mosquito larvae.

## Materials and methods

### Study site

Mosquito sampling was conducted at the Trelease Woods (40° 7′ 51.01′′ N; 88° 8′ 28.08′′ W) and South Farms (40° 5′ 6.59′′ N; 88° 12′ 57.79′′ W) study sites in Urbana, IL. Trelease Woods is a 71.17-acre deciduous forest situated ~ 9.5 km northeast of the University of Illinois at Urbana-Champaign (UIUC) campus. This is a woodland ecosystem consisting mainly of mature oak (*Quercus* spp.), ash (*Fraxinus* spp.), hackberry (*Celtis occidentalis*) and maple species (*Acer saccharinum* Linn.), with a high, closed canopy and moderately dense understory. The site includes two small seasonal ponds which provide suitable habitat for many aquatic and semi-aquatic invertebrates, including mosquitoes. South Farms is a 20.14-acre woodland approximately 2.41 km southeast of UIUC campus. It is composed of low canopy trees consisting mainly of silver maple (*Acer saccharinum* Linn.), Sycamore (*Platanus occidentalis* Linn.), pine (*Pinus* spp.), oak (*Quercus* spp.) and patchy grass undergrowth in some sections ^104^. Approximately 1 year prior to this study, the invasive Amur honeysuckle (*Lonicera maackii*), that was the dominant shrub in this woodlot was removed.

### Sampling and laboratory sample preparation

At South Farms, mosquitoes were sampled from ten used tires and five tree holes. Tree holes that were productive for mosquito larvae were randomly distributed in the study location, typically occurring approximately 10–20 m apart. In tree hole habitats, only *Ae. triseriatus* species were present, while both *Ae.triseriatus* and *Ae. japonicus* were present in used tire habitats. Since preliminary investigations at Trelease Woods did not yield any tree holes at a reachable level, sampling in this study site was restricted to ten used tires only, and both *Ae. triseriatus* and *Ae. japonicus* were detected and sampled from the used tire habitats. At the beginning of June 2016, used tires of equal sizes were established at both South Farms and Trelease Woods by securing them to the ground with metal rebar and zip ties at an angle of ~ 60° to enable colonization with detritus and filling with rainwater. Used tires were placed at least 20 m apart. To initiate these habitats, 4 L of tap water were added to each used tire and subsequently replenished naturally with rainwater. Sampling was initiated 2 weeks following the first rain event on 7 June 2016 and then weekly until 7 August 2016. During this period, it rained at least once every week, enough to replenish the habitats. Repeated sampling of each individual larval habitat was performed on 12 sampling dates within the entire sampling duration. Before drawing water for larval sampling in each habitat, water chemistry parameters (i.e. conductivity, pH, temperature, salinity, and total dissolved solids) were quantified using Extech pH/Conductivity, and Extech Dissolved Oxygen meters (Extech Instruments, MA), for dissolved oxygen measurements. Additionally, 50 mL and 40 mL water samples were collected into sterile 50 mL centrifuge tubes and transported to the lab for calorimetric assays, and bacterial community composition analysis, respectively. Water samples were collected from the habitats every mosquito sampling period whenever there was water and mosquitoes in the sampling habitats. Calorimetric assay of turbidity, tannin and lignin, reactive phosphorus and nitrogen ammonia, was conducted in the laboratory using Hach DR/800 Portable Calorimeter (Hach Company, Loveland, CO) according to the manufacturer’s instructions. Prior to collecting water for bacterial analysis, the contents of the larval habitat were initially agitated using a turkey baster by drawing in and out the water content repeatedly to mix them thoroughly. This was repeated at each sampling site. Following collection of the water samples, approximately 400 mL water samples were drawn from each tire and tree hole habitat using 40 mL-capacity turkey baster and transferred into 500 mL-capacity whirl Pak bags. The turkey basters were cleaned between each sampling interval. In the laboratory, water samples were individually emptied into clean 2 L enamel pans, and fourth instar larvae were sorted and identified to species level using morphological keys^105,106^ on a stereomicroscope. Late fourth instar larvae of *Ae. triseriatus* and *Ae. japonicus,* the most abundant species in all sample collections, were preserved in − 80 °C freezer until further processing.

### DNA extraction

Fourth instar larvae were retrieved from − 80 °C freezer, surface-sterilized individually in 70% ethanol for 10 min, five times in sterile water, and once in sterile 1× DPBS solution^8^. Individual whole larvae were suspended in bead solution of PowerSoil DNA Isolation Kit, homogenized using Retsch MM 300 TissueLyser (Retsch, Haan, Germany) and genomic DNA extracted using MoBio PowerSoil DNA Isolation Kit (MoBio Laboratories, Inc., CA) following the manufacturer’s protocol. The final DNA material from each sample was eluted in 100 µL of sterile elution buffer (10 mM Tris–HCl). DNA was quantified using Nanodrop 1000 (ThermoFisher Scientific, Pittsburgh, PA). Water samples for microbiome analysis were thawed at room temperatures and 30 mL of each sample centrifuged for 20 min at 5000 RPM using Eppendorf Centrifuge 5810R (ThermoFisher Scientific, Waltham, MA). The supernatant was discarded, and the pellets suspended in bead solution of PowerSoil DNA Isolation Kit and the DNA extracted following the same procedures used in the larval samples. The samples were shipped on Dry Ice to the National Center for Agricultural Utilization Research, Peoria, IL for sequencing. The V3–V4 hypervariable region of bacterial 16S rRNA gene was PCR-amplified using previously published universal primers, 341f, and 806r^107,108^. The V3–V4 hypervariable region of the 16S rRNA has been shown to have higher sensitivity in bacterial phylogenetic analysis compared to the rest of the hypervariable regions of the 16S rRNA gene^109^. The following primer set specific for V3–V4 region of the 16S rRNA gene were used: Forward 5′CCTACGGGNGGCWGCAG; Reverse 5′GACTACHVGGGTATCTAATCC. The primers were incorporated into fusion primers for dual indexing and incorporation of adapters prior to genome sequencing using Illumina MiSeq (Illumina Inc., San Diego, CA)^110^. There were two PCR cycles. The first PCR cycle was used to amplify the sequences using the primers targeting V3–V4 hypervariable region of 16S rRNA gene, using the following PCR conditions: 95 °C for 3 min; 25 cycles of: 95 °C for 30 s, 55 °C for 30 s, 72 °C for 30 s; 72 °C for 5 min; hold at 4 °C. The second PCR cycle was used to add the indexes using the following PCR conditions: 95 °C for 3 min; 8 cycles of: 95 °C for 30 s, 55 °C for 30 s, 72 °C for 30 s; 72 °C for 5 min; hold at 4 °C. A negative control sample consisting of DNA extracted from molecular grade water was sequenced with the same protocol to allow detection of any background contamination, but none was detected. PCR amplicons were cleaned and normalized using a SequalPrep normalization plate (Thermofisher Scientific, Waltham, MA). The samples were pooled, and the library quantified with a Kapa library quantification kit (Kapa Biosystems Willington, MA). The pooled library was mixed with Phix control spike-in of 5% as a sequencing control. The samples were sequenced using an Illumina MiSeq system with a MiSeq V3 2 × 300 bp sequencing kit. The demultiplexed reads were quality-trimmed to Q30 using CLC genomics workbench v8.5 (Qiagen Inc., Valencia, CA). Read pairing, fixed length trimming and operational taxonomic unit (OTU) clustering was done using CLC Bio Microbial Genomics module (Qiagen Inc., Valencia, CA) using the reference sequences from the Greengenes ribosomal RNA gene database and OTU assignment at 99% sequence similarity, which is adequate for bacterial identification to the genus level^111^.

### Statistical analysis

All analyses were conducted using R version 4.0.0^112^ within Rstudio environment version 1.3.959^113^ statistical package. Bacterial OTUs accounting for < 0.005% of the total number of sequences were removed from the analysis to reduce the problem of artifact OTUs^114^. From an initial sample size of 276 samples, 84 samples had < 1058 reads and were excluded from further analysis. The 1058 sampling depth was chosen to ensure it is high enough to capture the diversity in samples without eliminating many samples with low sequence counts that represent actual bacterial OTUs. Rarefaction curves also began leveling off at this cut-off point indicating a good compromise (Fig. S1). Sequence reads were rarefied to 1058 reads per sample to standardize library sizes. To estimate sample coverage, rarefaction curves were generated using unrarefied sequence data using the “phyloseq” package version 1.32.0 in R^115–117^. Alpha diversity metrics, including Shannon diversity index, observed species, and chao1, were generated in QIIME 2 (version 2019.10)^118^. Kruskal–Wallis test was used to test for differences in means between treatment combinations of sample type (*Ae. japonicus* vs. *Ae. triseriatus* vs. water samples) and sampling habitat, and pairwise Wilcoxon rank sum test with Bonferroni correction used to compute pairwise comparisons of the treatment groups. Beta-diversity measures were conducted using Bray–Curtis dissimilarity index using “phyloseq” package and non-metric multidimensional scaling (NMDS) ordination plots were generated to visualize the results. Permutational ANOVA (PERMANOVA) test was used to test for the main and interactive effect of sample type (*Ae. japonicus*, *Ae. triseriatus*, water samples) and individual habitat type on mosquito bacterial community composition^119^. PERMANOVA was conducted using the adonis function in the vegan package with 999 permutations^120^. PERMANOVA is sensitive to within-group variation, therefore, an analysis of multivariate homogeneity (PERMDISP) was conducted using the function betadisper to test for within-group dispersions with 999 permutations^119^. Post-hoc multiple pairwise comparisons using function “pairwise.adonis” in the vegan package^120^ was conducted to isolate individual sampling sites that differed significantly in bacterial community composition between *Ae. triseriatus*, *Ae. japonicus* and water samples. The analyses were conducted separately for each of the two study locations (South Farms or Trelease Woods). The *p*-values were Bonferroni-adjusted to reduce type-I error rate. Venn diagrams were created using the R package “limma”^121^ to visualize OTUs that were shared between the two mosquito species and water samples across habitat types and sampling locations. Canonical discriminant analysis (CDA) was used to classify the water chemistry parameters and the top 1% of the most abundant OTUs by habitat type. CDA separates groups by identifying the linear combination of variables having the highest multiple correlations within the groups^63^. Standardized canonical coefficients were used to evaluate the relative contribution of each predictor variable to the classification outcome. Prior to running the CDA analysis, multicollinearity diagnosis was done to remove correlated predictor variables and those with variance inflation factor (VIF) > 10 removed^122^. The analysis was performed in R package “candisc” version 0-8.3^123^ and the results visualized on CDA plots.

## Supplementary Information

Supplementary Information.
