# Supplementary material for: The larval environment strongly influences the bacterial communities of Aedes triseriatus and Aedes japonicus (Diptera: Culicidae)
Source: Sci Rep. 2021 Apr 12;11:7910. doi: 10.1038/s41598-021-87017-0 (PMC8042029; doi:10.1038/s41598-021-87017-0)
Supplement: Supplementary file 1 — Supplementary Information. [file 41598_2021_87017_MOESM1_ESM.docx]

**The larval environment strongly influences the bacterial communities of *Aedes triseriatus* and *Aedes japonicus* (Diptera: Culicidae)**

Elijah O. Juma^1*^, Brian F. Allan^1^, Chang-Hyun Kim^2^, Christopher Stone^2^, Christopher Dunlap^3^, Ephantus J. Muturi^3^

^1^Department of Entomology, University of Illinois at Urbana-Champaign, 505 S. Goodwin Ave, Urbana, IL 61801, USA

^2^Illinois Natural History Survey, University of Illinois at Urbana-Champaign, 1816 S. Oak St., Champaign, IL 61820, USA

^3^Crop Bioprotection Research Unit, Agricultural Research Service, U.S. Department of Agriculture, 1815 N. University St. Peoria IL 61604, USA

*Corresponding Author: 505 S. Goodwin Ave, Urbana, IL 61801, USA. Tel: +254 711 59 51 26; E-mail: [juma2@illinois.edu](mailto:juma2@illinois.edu); [elijahjuma@gmail.com](mailto:elijahjuma@gmail.com)


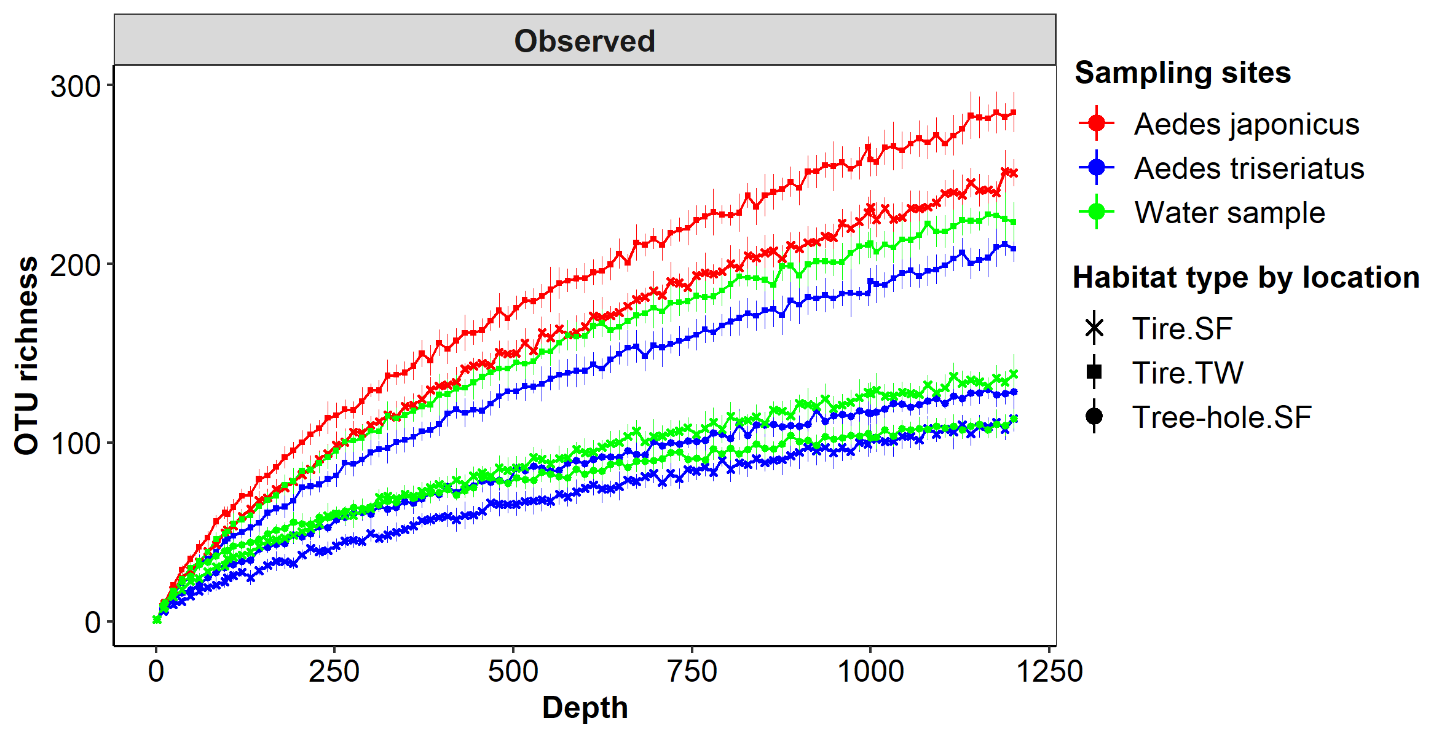
 **Figure S1** Rarefaction curves of OTU diversity for the mosquito and water sample combinations. Samples were pooled by mosquito or water sample type and rarefaction analysis done based on sample type by habitat type and study location. SF – South Farms; TW – Trelease Woods. Rarefaction curves represent the number of new OTUs discovered by sampling without replacement


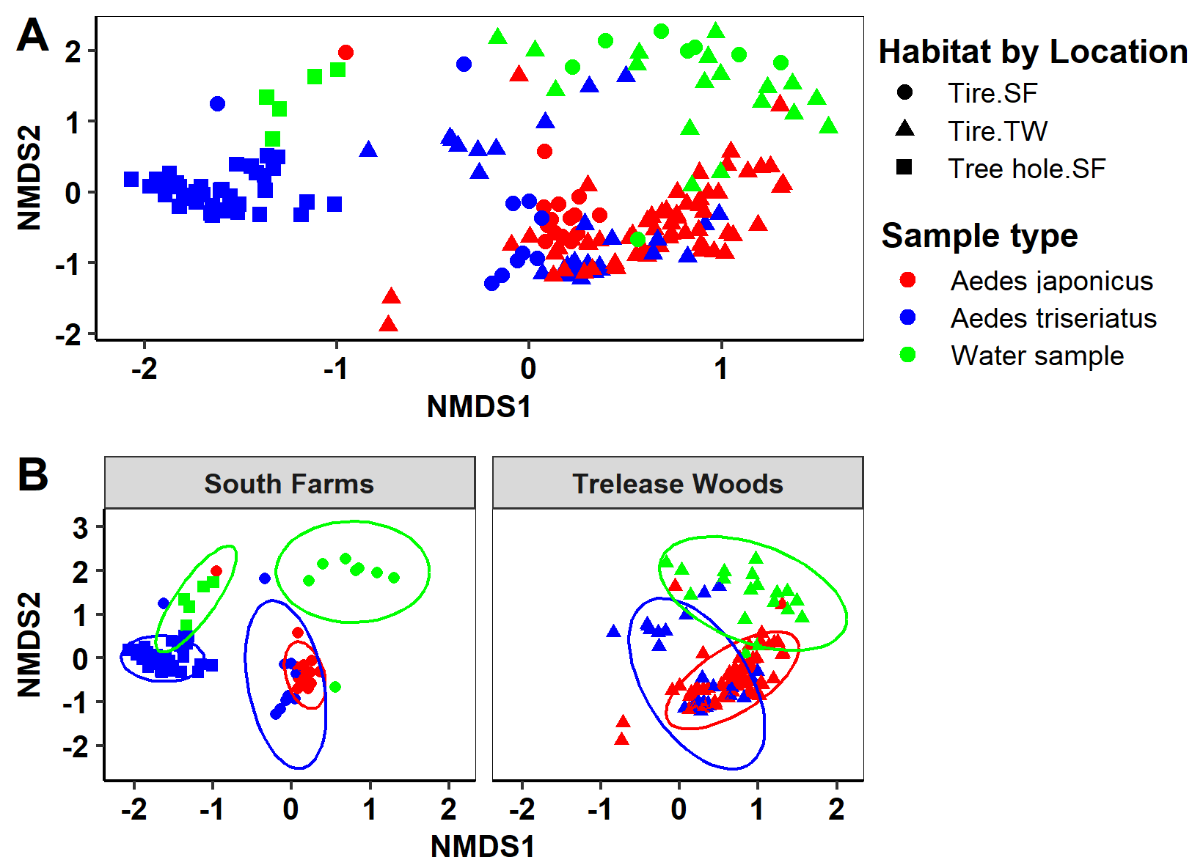


**Figure** **S2** NMDS using Bray-Curtis distance matrix comparing bacterial communities between two mosquito species and water samples; (A) combined plot; (B) bacterial OTU data partitioned by habitat type and sampling site. SF – South Farms; TW – Trelease Woods


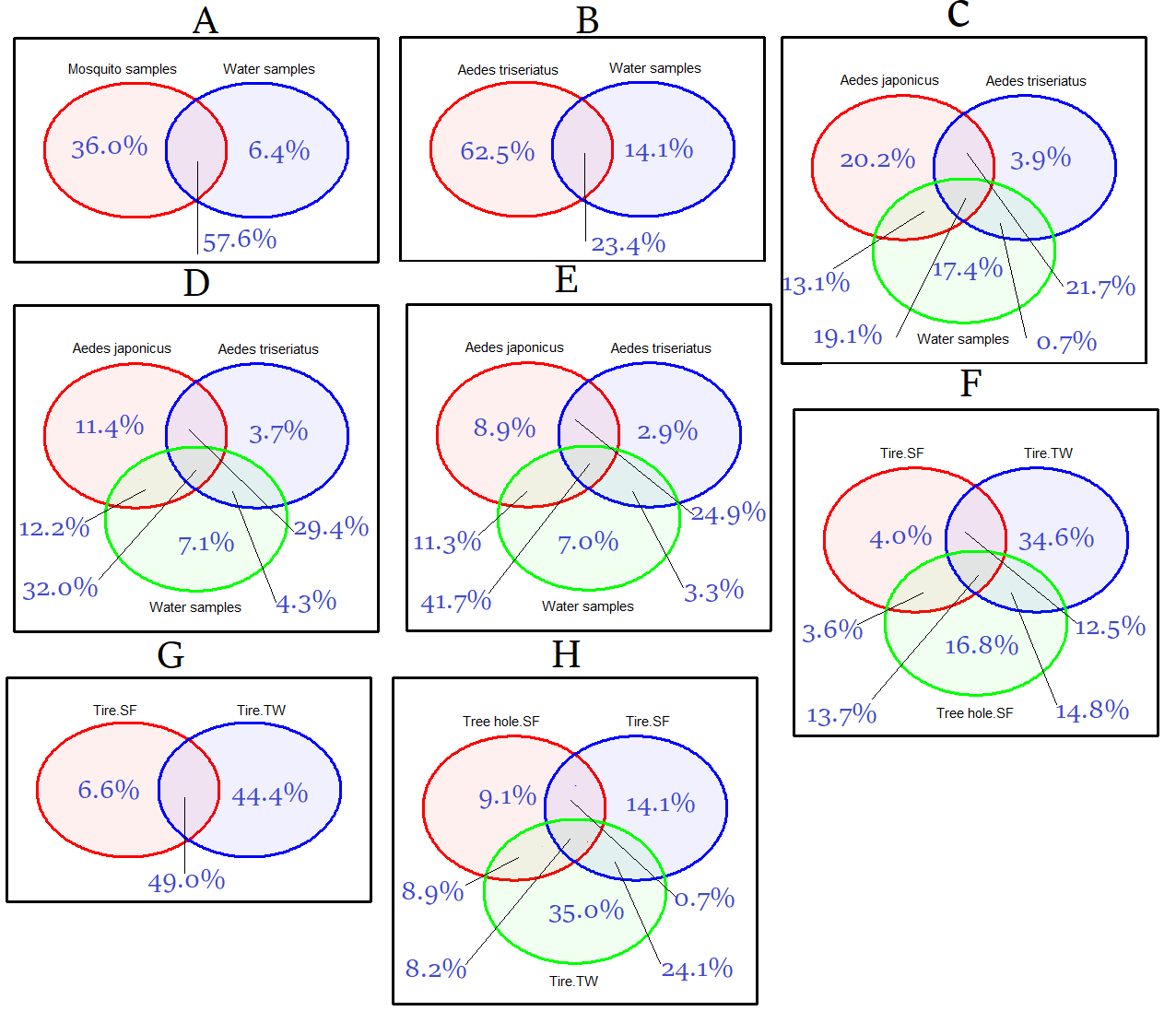


**Figure S3.** Venn diagram showing the number of unique and shared OTUs among different treatments of mosquito and water samples. The values each set represent the percentage of OTUs that are unique to each treatment. The values in the intersection represent the percentage of OTUs shared between treatments. (A) Unique and shared OTUs from pooled samples of mosquito and water samples; (B) unique and shared OTUs between mosquito and water samples from tree-holes at South Farms; (C) unique and shared OTUs between mosquito and water samples from used tires at South Farms; (D) unique and shared OTUs between mosquito and water samples from used tires at Trelease Woods; (E) unique and shared OTUs between mosquito and water samples for all pooled samples from used tires; (F) unique and shared OTUs in *Ae. triseriatus* partitioned by habitat type and study site; (G) unique and shared OTUs in *Ae. japonicus* partitioned by habitat type, and study site; (H) unique and share OTUs between water samples from tire and tree hole habitats from the two study sites (WS- water sample; TI – used tire, TH – tree hole). AT- *Aedes triseriatus*; AJ – *Aedes japonicus*; WS – water sample; TI – used tire; SF – South Farms; TW – Trelease Woods


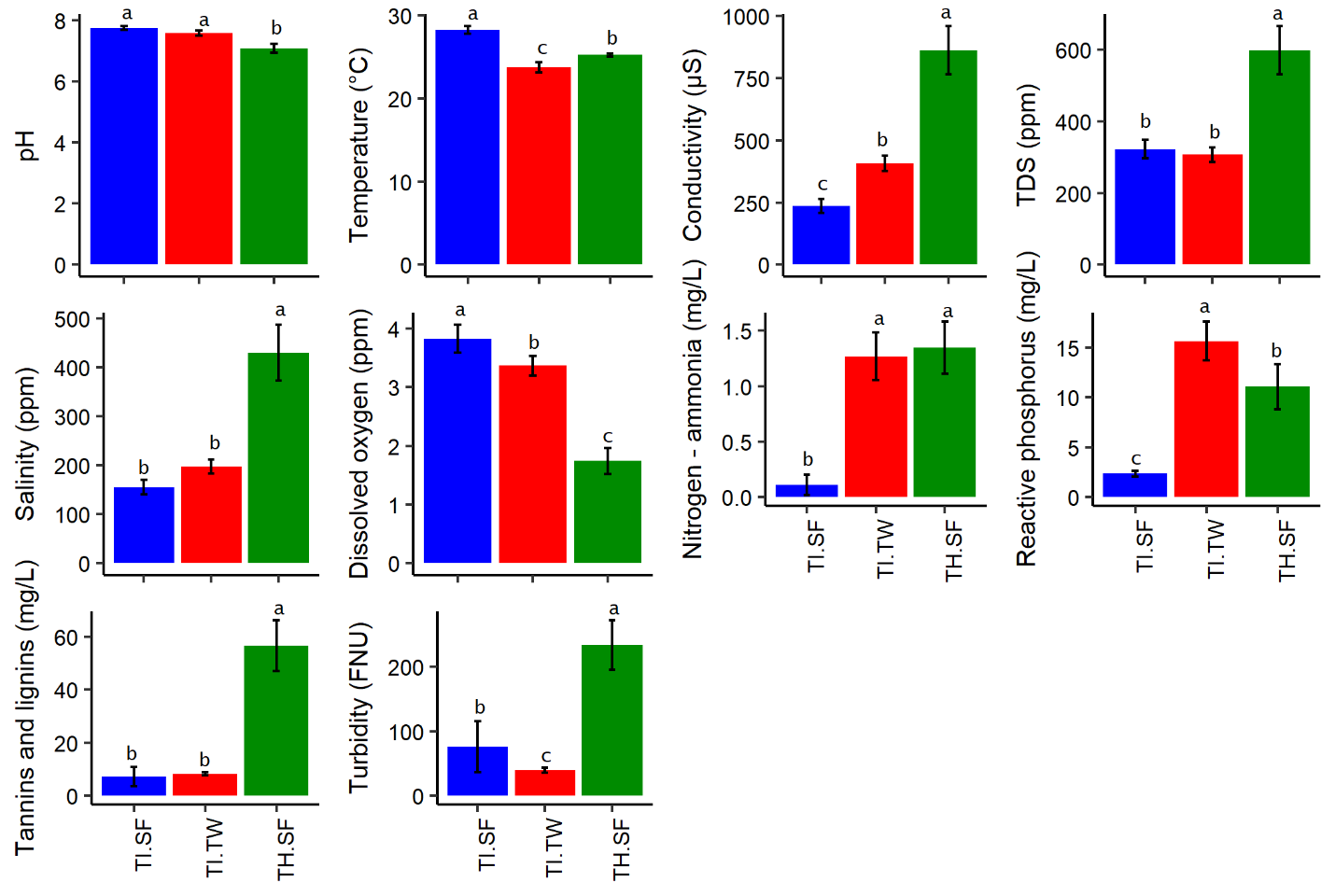


**Figure S4** Water chemistry parameters (mean±) recorded from used tires and tree-holes from South Farms and Trelease Woods. TI – used tires; TH – tree hole; TW – Trelease Woods SF – South Farms

**Table S1**. Bacterial diversity and richness (mean ± SE) for mosquito and water samples from individual sampling sites. Samples that occurred in the sampling site only once do not have mean ± SE values. AJ – *Aedes japonicus*; AT – *Aedes triseriatus*; TI – used tire; TH – tree hole; SF – South Farms; TW – Trelease Woods

| **Sampling sites** | **Observed OTUs** | **Chao1** | **Shannon** |
| --- | --- | --- | --- |
| AJ.TI.SF.1 | 151.75±41.12 | 189.90±0.93 | 4.78±0.93 |
| AJ.TI.SF.2 | 144.25±19.98 | 183.40±0.42 | 4.76±0.42 |
| AJ.TI.SF.3 | 191.43±16.50 | 240.64±0.25 | 4.68±0.25 |
| AJ.TI.TW.1 | 227.20±25.36 | 278.70±0.19 | 5.30±0.19 |
| AJ.TI.TW.2 | 225.75±33.94 | 284.03±0.29 | 5.42±0.29 |
| AJ.TI.TW.3 | 209.33±11.46 | 251.88±0.26 | 5.43±0.26 |
| AJ.TI.TW.4 | 127.00 | 195.33 | 5.19 |
| AJ.TI.TW.5 | 144.20±18.55 | 193.35±0.18 | 4.51±0.18 |
| AJ.TI.TW.6 | 174.22±17.45 | 207.53±0.39 | 4.96±0.39 |
| AJ.TI.TW.7 | 166.38±11.96 | 227.61±0.22 | 4.37±0.22 |
| AJ.TI.TW.8 | 136.00±20.65 | 175.32±0.22 | 4.41±0.22 |
| AJ.TI.TW.9 | 186.00±12.24 | 236.61±0.27 | 5.04±0.27 |
| AJ.TI.TW.10 | 174.00±17.75 | 235.48±0.15 | 4.32±0.15 |
| AT.TH.SF.1 | 157.71±6.76 | 197.83±9.84 | 4.34±0.21 |
| AT.TH.SF.2 | 185.33±3.93 | 248.16±6.31 | 3.92±0.55 |
| AT.TH.SF.3 | 160.40±8.08 | 212.22±13.75 | 3.00±0.12 |
| AT.TH.SF.4 | 131.89±5.33 | 175.82±6.87 | 3.41±0.12 |
| AT.TH.SF.5 | 111.89±4.71 | 145.36±6.46 | 3.49±0.30 |
| AT.TI.SF.1 | 149.50±18.51 | 217.38±11.76 | 3.13±0.23 |
| AT.TI.SF.2 | 76.00 | 170.50 | 3.14 |
| AT.TI.SF.3 | 154.33±25.05 | 230.16±50.86 | 4.37±0.06 |
| AT.TI.TW.1 | 166.00 | 248.45 | 3.10 |
| AT.TI.TW.3 | 132.00 | 171.14 | 4.12 |
| AT.TI.TW.4 | 112.20±8.63 | 156.63±12.24 | 3.57±0.19 |
| AT.TI.TW.6 | 184.29±5.91 | 229.05±8.45 | 4.69±0.20 |
| AT.TI.TW.10 | 197.60±13.43 | 263.29±13.53 | 4.34±0.20 |
| WS.TH.SF.1 | 76.00 | 113.50 | 4.01 |
| WS.TH.SF.2 | 76.00 | 97.00 | 4.13 |
| WS.TH.SF.3 | 120.00 | 212.81 | 3.68 |
| WS.TH.SF.4 | 148.00 | 254.94 | 4.58 |
| WS.TH.SF.5 | 78.00 | 105.27 | 4.15 |
| WS.TI.SF.1 | 115.67±23.69 | 157.20±28.12 | 4.06±0.74 |
| WS.TI.SF.2 | 90.00±8.00 | 136.38±33.38 | 3.44±0.35 |
| WS.TI.SF.3 | 108.00±10.60 | 175.41±32.09 | 3.72±0.08 |
| WS.TI.TW.1 | 125.50±3.50 | 192.86±6.36 | 4.04±0.51 |
| WS.TI.TW.2 | 142.00 | 273.40 | 1.87 |
| WS.TI.TW.3 | 165.33±16.50 | 249.91±29.80 | 3.78±0.14 |
| WS.TI.TW.4 | 118.50±17.50 | 176.79±34.46 | 3.94±0.12 |
| WS.TI.TW.6 | 158.00±57.66 | 223.02±75.55 | 3.73±1.25 |
| WS.TI.TW.7 | 208.67±32.32 | 293.81±61.18 | 3.50±0.88 |
| WS.TI.TW.8 | 137.00 | 180.05 | 3.79 |
| WS.TI.TW.9 | 151.50±5.50 | 205.58±7.53 | 4.68±0.28 |
| WS.TI.TW.10 | 133.00 | 251.46 | 5.00 |

**Table S2.** Multiple pairwise comparison of differences in bacterial community composition of mosquito larvae and water samples between mosquito species and water samples. Bacterial communities differed significantly between mosquito species and water samples after Bonferroni correction

| **Sampling type** | **Df** | **SS** | **F. Model** | **R^2^** | ***P.* value** | ***P*. adjusted** |
| --- | --- | --- | --- | --- | --- | --- |
| *Aedes triseriatus* vs *Aedes japonicus* | 1 | 7.22 | 19.68 | 0.11 | 0.001 | 0.003 |
| *Aedes triseriatus* vs Water sample | 1 | 4.92 | 13.25 | 0.11 | 0.001 | 0.003 |
| *Aedes japonicus* vs Water sample | 1 | 4.03 | 10.46 | 0.09 | 0.001 | 0.003 |

**Table S3** Multiple pairwise comparison of group mean differences in bacterial community composition of *Ae. japonicus, Ae. triseriatus* and water samples at the individual sampling sites, at South Farms study location. Bacterial composition differed significantly between *Ae. japonicus, Ae. triseriatus* and water samples in majority of the individual sampling sites after Bonferroni correction of the *p* values. TH – tree hole; TI – used tire

| **Sampling sites** | **Df** | **Sums of Sqs** | **F. Model** | **R^2^** | ***P*. value** | ***P.* adjusted** |
| --- | --- | --- | --- | --- | --- | --- |
| TH.5 vs TH.3 | 1 | 0.633526924 | 6.059415 | 0.241802 | 0.001 | 0.028 |
| TH.5 vs TH.4 | 1 | 0.798269922 | 6.835257 | 0.275224 | 0.001 | 0.028 |
| TH.5 vs TI.1 | 1 | 3.323180516 | 14.552 | 0.409316 | 0.001 | 0.028 |
| TH.5 vs TH.1 | 1 | 1.053760947 | 8.349737 | 0.342909 | 0.001 | 0.028 |
| TH.5 vs TI.2 | 1 | 2.399621323 | 11.81376 | 0.440586 | 0.001 | 0.028 |
| TH.5 vs TI.3 | 1 | 3.610612583 | 17.65516 | 0.456735 | 0.001 | 0.028 |
| TH.3 vs TH.4 | 1 | 1.228423315 | 11.13574 | 0.369519 | 0.001 | 0.028 |
| TH.3 vs TI.1 | 1 | 3.463366829 | 15.90866 | 0.419658 | 0.001 | 0.028 |
| TH.3 vs TH.1 | 1 | 1.453086587 | 12.27125 | 0.419225 | 0.001 | 0.028 |
| TH.3 vs TI.2 | 1 | 2.582277889 | 13.58823 | 0.459245 | 0.001 | 0.028 |
| TH.3 vs TI.3 | 1 | 3.743087582 | 19.20227 | 0.466049 | 0.001 | 0.028 |
| TH.4 vs TI.1 | 1 | 3.240337461 | 13.8726 | 0.397808 | 0.001 | 0.028 |
| TH.4 vs TH.1 | 1 | 1.41535889 | 10.63826 | 0.39936 | 0.001 | 0.028 |
| TH.4 vs TI.2 | 1 | 2.387822062 | 11.34798 | 0.430696 | 0.001 | 0.028 |
| TH.4 vs TI.3 | 1 | 3.490338395 | 16.64285 | 0.442125 | 0.001 | 0.028 |
| TI.1 vs TH.1 | 1 | 2.738891448 | 10.79149 | 0.362234 | 0.001 | 0.028 |
| TI.1 vs TI.3 | 1 | 1.695099502 | 5.731825 | 0.192784 | 0.001 | 0.028 |
| TI.1 vs TH.2 | 1 | 1.487262982 | 4.969236 | 0.248845 | 0.001 | 0.028 |
| TH.1 vs TI.3 | 1 | 2.887556136 | 12.69638 | 0.400563 | 0.001 | 0.028 |
| TI.2 vs TI.3 | 1 | 1.308656518 | 4.403973 | 0.196571 | 0.001 | 0.028 |

| TH.1 vs TI.2 | 1 | 1.979383075 | 8.372608 | 0.391745 | 0.002 | 0.056 |
| --- | --- | --- | --- | --- | --- | --- |
| TI.2 vs TH.2 | 1 | 1.239176844 | 4.069481 | 0.311373 | 0.002 | 0.056 |
| TI.3 vs TH.2 | 1 | 1.642535002 | 6.17744 | 0.291699 | 0.002 | 0.056 |
| TH.4 vs TH.2 | 1 | 0.546456159 | 3.651363 | 0.233294 | 0.011 | 0.308 |
| TI.1 vs TI.2 | 1 | 1.018064226 | 3.132618 | 0.148236 | 0.012 | 0.336 |
| TH.3 vs TH.2 | 1 | 0.539332338 | 4.172842 | 0.242991 | 0.013 | 0.364 |
| TH.1 vs TH.2 | 1 | 0.461732633 | 2.695501 | 0.212319 | 0.048 | 1 |
| TH.5 vs TH.2 | 1 | 0.286219281 | 2.03662 | 0.145093 | 0.08 | 1 |

|  |  |  |  |  |  |  |
| --- | --- | --- | --- | --- | --- | --- |
|  |  |  |  |  |  |  |
|  |  |  |  |  |  |  |
|  |  |  |  |  |  |  |
|  |  |  |  |  |  |  |
|  |  |  |  |  |  |  |

**Table S4** Multiple pairwise comparison of group mean differences in bacterial community composition of *Ae. japonicus, Ae. triseriatus* and water samples at the individual sampling sites, at Trelease Woods study location. Bacterial composition differed significantly between *Ae. japonicus, Ae. triseriatus* and water samples in majority of the individual sampling sites after Bonferroni correction of the *p* values. TI – used tire

| **Sampling sites** | **Df** | **Sums of Sqs** | **F. Model** | **R^2^** | ***P.* value** | ***P.* adjusted** |
| --- | --- | --- | --- | --- | --- | --- |
| TI.6 vs TI.1 | 1 | 1.667095237 | 5.93686 | 0.156493 | 0.001 | 0.036 |
| TI.6 vs TI.8 | 1 | 2.02196085 | 7.96129 | 0.215395 | 0.001 | 0.036 |
| TI.6 vs TI.4 | 1 | 3.366519316 | 12.42779 | 0.251433 | 0.001 | 0.036 |
| TI.6 vs TI.3 | 1 | 1.269102275 | 4.515969 | 0.127153 | 0.001 | 0.036 |
| TI.6 vs TI.7 | 1 | 3.871531018 | 14.34862 | 0.264011 | 0.001 | 0.036 |
| TI.6 vs TI.9 | 1 | 3.590369115 | 12.96603 | 0.244799 | 0.001 | 0.036 |
| TI.6 vs TI.10 | 1 | 3.284306201 | 14.26454 | 0.267806 | 0.001 | 0.036 |
| TI.6 vs TI.2 | 1 | 1.345775555 | 5.252156 | 0.153338 | 0.001 | 0.036 |
| TI.1 vs TI.4 | 1 | 1.47063981 | 4.631168 | 0.195977 | 0.001 | 0.036 |
| TI.1 vs TI.9 | 1 | 1.117639829 | 3.469492 | 0.136221 | 0.001 | 0.036 |
| TI.1 vs TI.10 | 1 | 1.621012773 | 6.821663 | 0.245192 | 0.001 | 0.036 |
| TI.1 vs TI.2 | 1 | 1.014020581 | 3.241129 | 0.227589 | 0.001 | 0.036 |
| TI.8 vs TI.4 | 1 | 1.615367552 | 5.856743 | 0.26796 | 0.001 | 0.036 |
| TI.8 vs TI.7 | 1 | 1.721333134 | 6.310139 | 0.249313 | 0.001 | 0.036 |
| TI.4 vs TI.3 | 1 | 1.467231791 | 4.585453 | 0.203027 | 0.001 | 0.036 |
| TI.4 vs TI.7 | 1 | 2.724543518 | 9.382361 | 0.257882 | 0.001 | 0.036 |
| TI.4 vs TI.9 | 1 | 2.487630212 | 8.267606 | 0.234425 | 0.001 | 0.036 |
| TI.4 vs TI.10 | 1 | 3.432446328 | 14.80673 | 0.36285 | 0.001 | 0.036 |
| TI.4 vs TI.2 | 1 | 1.387247899 | 4.956091 | 0.236499 | 0.001 | 0.036 |
| TI.3 vs TI.7 | 1 | 1.217215874 | 3.914762 | 0.157126 | 0.001 | 0.036 |
| TI.3 vs TI.10 | 1 | 1.122547049 | 4.76036 | 0.192257 | 0.001 | 0.036 |
| TI.7 vs TI.9 | 1 | 2.438560484 | 8.225443 | 0.215182 | 0.001 | 0.036 |
| TI.7 vs TI.10 | 1 | 3.595690609 | 15.34088 | 0.345976 | 0.001 | 0.036 |
| TI.7 vs TI.2 | 1 | 1.430620917 | 5.178973 | 0.214193 | 0.001 | 0.036 |
| TI.9 vs TI.10 | 1 | 2.30629491 | 9.445795 | 0.245691 | 0.001 | 0.036 |
| TI.9 vs TI.2 | 1 | 1.529080629 | 5.25176 | 0.216552 | 0.001 | 0.036 |
| TI.10 vs TI.2 | 1 | 1.983098911 | 10.3912 | 0.366001 | 0.001 | 0.036 |
| TI.1 vs TI.7 | 1 | 1.077294518 | 3.483587 | 0.136699 | 0.002 | 0.072 |
| TI.8 vs TI.10 | 1 | 1.221609729 | 6.525532 | 0.266071 | 0.003 | 0.108 |
| TI.3 vs TI.9 | 1 | 0.992271353 | 3.058523 | 0.127128 | 0.005 | 0.18 |
| TI.3 vs TI.2 | 1 | 0.991033432 | 3.128737 | 0.238312 | 0.008 | 0.288 |
| TI.8 vs TI.2 | 1 | 1.354915242 | 5.952483 | 0.426625 | 0.009 | 0.324 |
| TI.1 vs TI.8 | 1 | 0.854034634 | 2.782735 | 0.2019 | 0.012 | 0.432 |
| TI.8 vs TI.3 | 1 | 0.736433309 | 2.374053 | 0.191857 | 0.022 | 0.792 |
| TI.1 vs TI.3 | 1 | 0.633840533 | 1.744824 | 0.118335 | 0.054 | 1 |
| TI.8 vs TI.9 | 1 | 0.439413093 | 1.527289 | 0.074403 | 0.158 | 1 |

**Table S5.** Results of canonical discriminant function analysis of the relationship between water chemistry parameters and the type of larval environment (used tires at South Farms; used tires at Trelease Woods, and tree holes at South Farms)

|  | **Discriminant function** | |  |
| --- | --- | --- | --- |
| **Water chemistry parameter** | **Can1** | **Can2** | ***P*** |
| _pH_ | _-0.44_ | _0.36_ | _<0.0001_ |
| _Temperature_ | _-0.48_ | _-1.10_ | _<0.0001_ |
| _Dissolved oxygen_ | _-0.62_ | _-0.27_ | _<0.0001_ |
| _Nitrogen ammonia_ | _0.01_ | _1.05_ | _<0.0001_ |
| _Reactive phosphorus_ | _0.08_ | _0.32_ | _<0.0001_ |
| _Tannin and lignin_ | _0.85_ | _-0.19_ | _<0.0001_ |
| _Turbidity_ | _-0.35_ | _-0.59_ | _<0.0001_ |
| _Summary statistics_ |  |  |  |
| _Wilks' Lambda_ | _0.06_ | _0.26_ |  |
| _Chi-square_ | _528.13_ | _247.48_ |  |
| _P value_ | _<0.0001_ | _<0.0001_ |  |
| _Eigen values_ | _3.67_ | _2.90_ |  |
| _Canonical correlation_ | _0.89_ | _0.86_ |  |
|  |  |  |  |

**Table S6**. Results of canonical discriminant function analysis of the relationship between the top 10 dominant bacterial OTUs in water samples and the larval environment type (used tires at South Farms; used tires at Trelease Woods, and tree holes at South Farms

|  | **Discriminant function** | |  |
| --- | --- | --- | --- |
| **Dominant bacterial OTUs** | **Can1** | **Can2** | ***P*** |
| _Dysgonomonas_ | _0.45_ | _-0.22_ | _0.07_ |
| *_Mycobacterium_* | _0.06_ | _-0.19_ | _0.89_ |
| *_Rhizobiales_* | _0.03_ | _0.49_ | _0.57_ |
| _Carnobacterium_ | _0.16_ | _0.49_ | _0.42_ |
| *_Comamonadaceae_* | _-0.78_ | _-0.18_ | _0.00_ |
| *_Sanguibacter_* | _0.24_ | _0.31_ | _0.39_ |
| _Actinomycetales_ | _0.22_ | _-0.11_ | _0.54_ |
| *_Burkholderiales_* | _0.06_ | _-0.41_ | _0.66_ |
| _Intrasporangiaceae_ | _0.12_ | _0.09_ | _0.81_ |
| _Leptothrix mobilis_ | _-0.22_ | _0.58_ | _0.25_ |
| _Summary statistics_ |  |  |  |
| _Wilk's Lambda_ | _0.85_ | _0.98_ |  |
| _Chi-square_ | _30.5_ | _4.5_ |  |
| _P value_ | _0.06_ | _0.87_ |  |
| _Eigen values_ | _0.15_ | _0.03_ |  |
| _Canonical correlation_ | _0.36_ | _0.16_ |  |
